# Modelling the impact of physical activity on MASLD in Saudi Arabia

**DOI:** 10.1371/journal.pone.0354332

**Published:** 2026-09-15

**Authors:** Carolyn Wallace, Saleh A. Alessy, Faisal Abaalkhail, Saad Alghamdi, Assim A. Alfadda, Abdulrahman Al Qabbani, Fadiah Alkhattabi, Reem S. AlAhmed, Tagreed A. Mazi, Hamdan Alghamdi, Sadeem Aldarwesh, Shoug Alashmali, Rowaedh A. Bawaked, Khalid Alobthani, Bandar Al-Judaibi, Waleed K. Al-Hamoudi, Dimitri A. Raptis, Brandon Eurich, Homie Razavi, Predrag Zivotic, Severin Rakic, Volkan Cetinkaya, Saleh A. Alqahtani

**Affiliations:** 1 Center for Disease Analysis Foundation, Lafayette, Colorado, United States of America; 2 Department of Public Health, Saudi Electronic University, Riyadh, Saudi Arabia; 3 Liver, Digestive, and Lifestyle Health Research Section, King Faisal Specialist Hospital and Research Center, Riyadh, Saudi Arabia; 4 Center for Cancer, Society and Public Health, Faculty of Life Sciences and Medicine, King’s College London, London, United Kingdom; 5 Department of Medicine, Gastroenterology Section, King Faisal Specialist Hospital and Research Center, Riyadh, Saudi Arabia; 6 College of Medicine, Alfaisal University, Riyadh, Saudi Arabia; 7 Organ Transplant Center of Excellence, King Faisal Specialist Hospital & Research Center, Riyadh, Saudi Arabia; 8 Obesity Research Center, and the Department of Medicine, College of Medicine, King Saud University, Riyadh, Saudi Arabia; 9 Department of Medicine, Section of Endocrinology, King Faisal Specialist Hospital and Research Center, Riyadh, Saudi Arabia; 10 Department of Pediatrics, King Faisal Specialist Hospital and Research Center, Riyadh, Saudi Arabia; 11 Department of Community Health Sciences – Clinical Nutrition, College of Applied Medical Sciences, King Saud University, Riyadh, Saudi Arabia; 12 Hepatology Section, Hepatobiliary Sciences and Organs Transplant Center, King Abdulaziz Medical City, Riyadh, Saudi Arabia; 13 King Saud Bin Abdulaziz University for Health Sciences, Ministry of National Guard Health Affairs, Riyadh, Saudi Arabia; 14 King Abdullah International Research Center, Ministry of National Guard Health Affairs, Riyadh, Saudi Arabia; 15 Department of Clinical Nutrition, Faculty of Applied Medical Sciences, King Abdulaziz University, Jeddah, Saudi Arabia; 16 Physical Rehabilitation Department, King Faisal Specialist Hospital and Research Center, Riyadh, Saudi Arabia; 17 Department of Medicine, Liver Disease Research Center, College of Medicine, King Saud University, Riyadh, Saudi Arabia; 18 World Bank Group, Washington, District of Columbia, United States of America; 19 Division of Gastroenterology and Hepatology, Weill Cornell Medicine, New York, United States of America; King Fahad Medical City, SAUDI ARABIA

## Abstract

**Background and aims:**

Insufficient physical activity contributes to the global rise of metabolic dysfunction–associated steatotic liver disease (MASLD). Although physical activity levels in Saudi Arabia have increased in recent years, MASLD shows an increasing prevalence trend, affecting over 30% of the population. This study aimed to determine the impact of increased physical activity, and resulting reduction in hepatic steatosis, on the projected health and economic burden of MASLD in Saudi Arabia.

**Methods:**

We simulated MASLD progression from 2025 to 2045 using a validated Markov model. Three aspirational physical activity intervention scenarios were evaluated, assuming that 10%, 20%, and 30% of individuals with MASLD achieved a clinically significant ≥30% relative reduction in hepatic steatosis. Health outcomes were measured in disability-adjusted life years (DALYs), and economic outcomes were analyzed using U.S.-based direct healthcare costs.

**Results:**

MASLD prevalence in Saudi Arabia is projected to increase from approximately 10.0 million in 2025 to 14.3 million in 2045. In scenarios where 10%, 20%, and 30% of individuals with MASLD achieved a ≥ 30% reduction in hepatic steatosis, cumulative liver-related deaths are projected to decline by 4.3%–12.3%, hepatocellular carcinoma cases by 4.9%–14.2%, and decompensated cirrhosis cases by up to 14.0%. Direct health care costs are estimated to reduce by 4.0%–11.5%, and DALYs are projected to decline by 8.6%–24.2% across scenarios.

**Conclusions:**

Increased physical activity could significantly reduce the future health and economic impacts of MASLD in Saudi Arabia. Achieving a ≥ 30% reduction in hepatic steatosis among individuals with MASLD could reduce liver-related complications, deaths, DALYs, and healthcare costs. These findings support the integration of MASLD-specific outcomes into national physical activity promotion campaigns and noncommunicable disease advocacy efforts, aligned with Saudi Vision 2030.

## Introduction

Obesity is a growing public health problem in Saudi Arabia, with over 30% of the population classified as obese [1,2]. Over the past three decades, obesity prevalence has more than doubled [3], in part due to lifestyle factors, including sedentary behaviors, dietary shifts, and rapid urbanization. The prevalence of metabolic dysfunction-associated steatotic liver disease (MASLD) is escalating alongside the obesity epidemic and is associated with substantial comorbidity, affecting more than 30% of the global population and a similarly high proportion of over 40% among adults in Saudi Arabia [4–11]. In response, the Kingdom’s Vision 2030 Health Sector Transformation Program has prioritized disease prevention, including initiatives to increase physical activity [12], which could reduce the related health and economic burden [13]. Multiple sectors have implemented over 40 policies and initiatives targeting physical activity, since its launch in 2016 [14,15]. Currently, 58% of the adult population in Saudi Arabia engages in adequate physical activity, which represents a significant progress in comparison to the previous decade [16,17]. Health benefits from increasing physical activity extend beyond weight reduction [18,19] to include reductions in hepatic steatosis, often independent of weight loss [20,21]. With approximately three-quarters of individuals with MASLD lacking adequate physical activity globally [22,23], there is a critical opportunity to reduce MASLD burden by increasing population-level physical activity [24].

As Saudi Arabia intensifies its national physical activity initiatives, the analysis sought to answer the research question: What could be the impact of increased physical activity on the future health and economic burden of MASLD in Saudi Arabia? We modelled multiple scenarios in which increased physical activity led to a reduction in hepatic steatosis, with the goal of quantifying potential improvements in disease outcomes and health care costs. The findings may provide actionable insights for the development of future national strategies aimed at reducing the prevalence of MASLD and its associated economic burden by promoting physical activity.

## Methods

**Fig 1:**
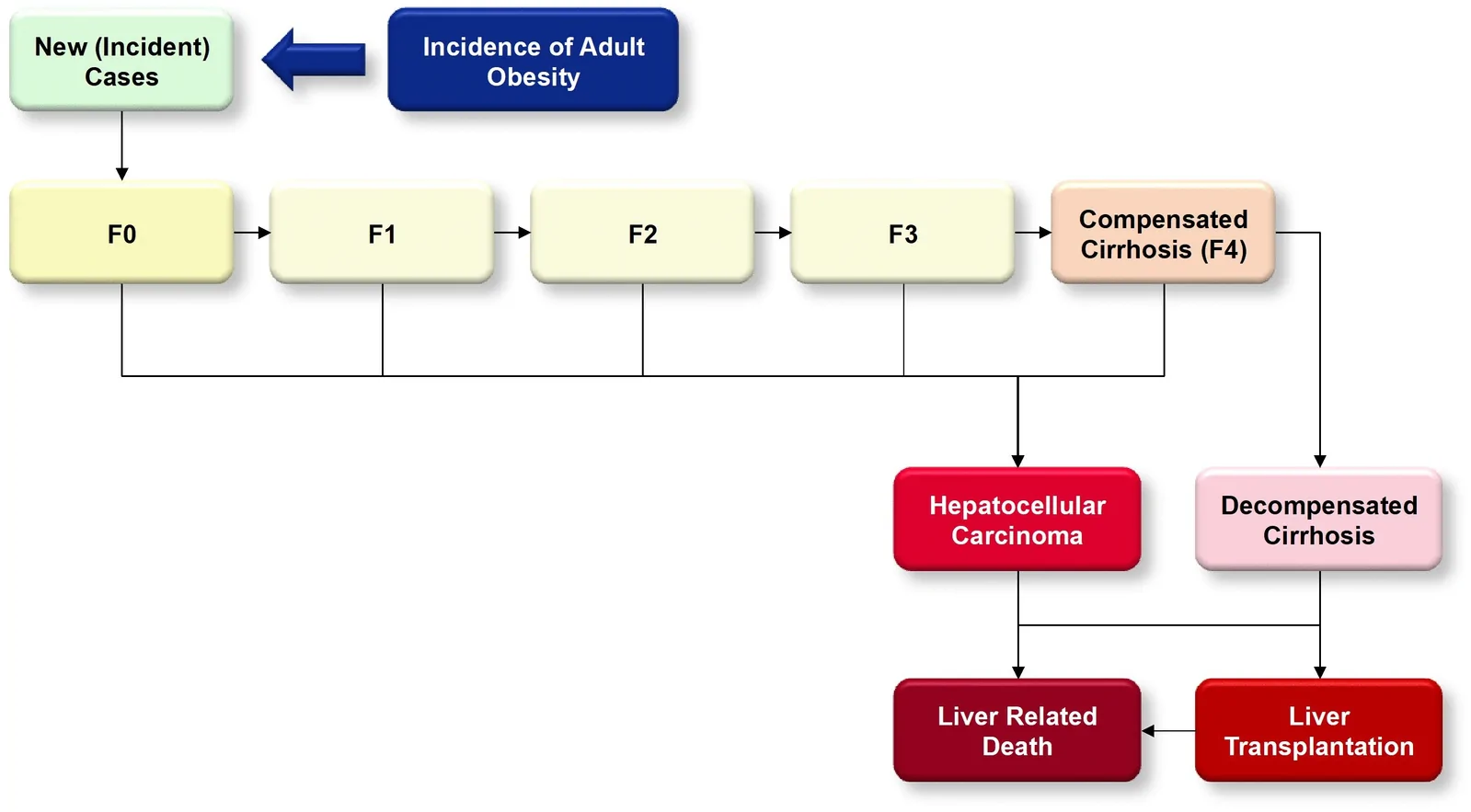
MASLD model disease progression flowchart. A Markov model is used to simulate disease progression in patients with MASLD, as presented in the flowchart. The flowchart starts from adult obesity and incorporates the progression of MASLD through several fibrosis stages, with each of them potentially leading to advanced diseases.

A Markov model was developed to simulate disease progression in patients with MASLD in Saudi Arabia from 2025 to 2045, estimating population size at each disease stage by age and sex (Fig 1). The model incorporated MASLD prevalence, progression rates, and liver-related mortality to comprehensively account for disease burden. Because MASLD and obesity are interrelated components of metabolic syndrome, trends in adult obesity prevalence were used to determine incident MASLD cases. The model was calibrated using reported prevalence estimates and validated with occurrences of advanced disease (hepatocellular carcinoma [HCC] and liver transplantation), for which accurate surveillance data were available. Progression rates were assumed to reflect the sum of forward progression minus the rate of regression, consistent with studies of consecutive liver biopsies [25]. It was conservatively assumed that the number of liver transplants in the future would not exceed current levels due to limited transplant availability, concerns related to MASH recurrence, and obesity-related comorbidities affecting outcomes [26]. Since patients with metabolic dysfunction-associated steatohepatitis (MASH) were assumed to experience increased all-cause mortality, the model applied a mortality multiplier based on reported standard mortality ratios and expert consensus. Although outcomes for MASH were not reported separately, its influence was incorporated into the model structure through elevated mortality and progression assumptions. Data quantifying the size of the comorbid MASLD and diabetes population was also used in validation steps. Model inputs were based on literature review, national data reports, and expert consensus. Details of the model were previously published [6] and are included in the S1 File. Model inputs and sources are listed in Table 1 and a minimum dataset underlying the results is included in the S2 File. Through this process, a status quo scenario was generated to forecast MASLD burden without additional interventions.

**Table 1:** Model Inputs and Sources.

Category	Item	Value	Source
Disease burden	Adult obesity prevalence	20.2% − 2019 23.1% − 2024	[49–51]
	MASLD prevalence by age and gender	–	[6,52]
	Fibrosis progression rates	–	[53–56]
	HCC incidence	2,520 2014-2020	[57–59]
	Total liver transplants	–	[60]
	% MASLD related	20-42% 2011-2023	[7]
	Type 2 Diabetes and MASLD	Diabetes w/ MASLD = 80.8% MASLD w/ Diabetes = 36.3%	[61,62]
Annual health care costs (2025 USD)	Compensated cirrhosis (prevalent)	$2,837	[63]
	Decompensated cirrhosis (prevalent)	$33,604	[63]
	Hepatocellular carcinoma (prevalent)	$53,602	[63]
	Liver transplant (incident)	$212,795	[63]
	Post-liver transplant (prevalent)	$46,345	[63]

HCC, hepatocellular carcinoma; MASLD, metabolic dysfunction–associated steatotic liver disease; USD, United States dollars.

A ≥ 30% relative reduction in hepatic steatosis correlates with improved disease status (reduced steatosis and fibrosis) [27,28] and can be achieved through physical activity independent of weight loss. In a pooled analysis, Stine et al. (2023) reported that 34% of participants in exercise training trials achieved a ≥ 30% relative reduction in hepatic steatosis, 21% more than control subjects [21]. In another pooled analysis of 3 randomized controlled trials (RCTs), 34% more participants in the aerobic exercise group than in control group achieved this degree of hepatic steatosis reduction [29]. Therefore, to model the impact of physical activity on MASLD, we investigated three aspirational scenarios in which 10%, 20%, and 30% of the population with MASLD achieved the clinically significant threshold of ≥30% relative reduction in hepatic steatosis.

Multiple studies, including a meta-analysis of 7 RCTs, concluded that approximately one-third more individuals with a ≥ 30% relative reduction in hepatic steatosis demonstrated MASH resolution and/or ≥2 point improvement in NAFLD Activity Score compared to those not meeting this threshold [27,28,30]. To reflect this, disease progression rates were uniformly adjusted for fibrosis stages F0-F3 for the portion of the MASLD population achieving this reduction (10% / 20% / 30% depending on scenario), resulting in one-third of these individuals in each stage achieving a halting of disease progression annually. It was assumed that reducing hepatic steatosis through physical activity would have minimal effect on halting disease progression in those with cirrhosis or more advanced liver disease, due to difficulty in reversing advanced fibrosis (see S1 File for adjusted progression rates).

It is well recognized that physical activity is effective in preventing weight gain [31] and lowering the risk of developing obesity. Using quantifiable and verifiable step count data and comparing the 75^th^ percentile to the 25^th^ percentile, a 40% risk reduction has been observed [18,32]. It is also established that physical activity lowers the risk of MASLD. A UK Biobank study concluded the risk of developing MASLD decreased by 47% with an increase in 2,500 steps per day [33]. Although not measuring incidence, a cross-sectional analysis of those meeting the guidelines of ≥150 minutes per week of physical activity were 44% less likely to have MASLD [34]. Therefore, in each scenario, the risk of developing MASLD was reduced by 40% among the subset of the population modeled to increase physical activity (10%, 20% or 30% scenarios).

To forecast the economic burden of MASLD, direct health care costs for patients with moderate-to-advanced disease stages were incorporated in the model. Published U.S.-based estimates of direct health care costs by disease stage (cirrhosis, decompensated cirrhosis [DCC], HCC, and liver transplantation) were used because comprehensive Saudi Arabia–specific cost estimates by MASLD disease stage were not available in the published literature. These estimates were adjusted for medical inflation [35], and a 3% annual discount rate was applied. Although absolute costs may differ between the U.S. and Saudi healthcare systems, the stage-specific cost structure provides a useful framework for estimating the relative economic burden associated with disease progression and the potential cost savings attributable to physical activity interventions. Disability-adjusted life years (DALYs) [36] were calculated using the Global Burden of Disease disability weights [37] and gross domestic product per capita for Saudi Arabia [38]. Economic inputs were held constant across scenarios to ensure consistency and isolate the effect of physical activity on the economic burden of MASLD. Health and economic outcomes were analyzed from 2025 to 2045.

### Statistical analysis

Given the ambiguous nature of reported data for MASLD populations, a sensitivity analysis was performed on key model inputs, including MASLD prevalence, transition rates, incidence reductions attributable to physical activity, the factor by which transition rates were affected by physical activity, and direct health care costs by disease stages (Table 2). Transition rate ranges were derived from published literature [25,39,40]. Annual healthcare costs were varied by ±20% in probabilistic sensitivity analyses to account for uncertainty associated with the transferability of U.S.-based cost estimates to the Saudi healthcare setting. Probabilistic sensitivity analysis was performed using Beta-PERT distributions in Oracle Crystal Ball (Oracle Corp., Redwood City, CA, Release 11.1.3708.0) to generate 95% uncertainty intervals.

**Table 2:** Uncertainty analysis parameters.

Key Model Inputs Assumptions*	Base	Low	High
2017 prevalence	25.7%	21%	31%
F0 to F1 transition probability	1.18%	0.70%	1.81%
F1 to F2 transition probability	7.27%	4.28%	11.14%
F2 to F3 transition probability	7.27%	4.28%	11.14%
F3 to F4 transition probability	7.05%	4.02%	13.40%
F4 to DCC transition probability	3.71%	2.60%	5.03%
F0 to HCC transition probability	0.0004%	0.0003%	0.0006%
F1 to HCC transition probability	0.008%	0.006%	0.011%
F2 to HCC transition probability	0.02%	0.013%	0.023%
F3 to HCC transition probability	0.03%	0.025%	0.045%
F4 to HCC transition probability	0.38%	0.29%	0.51%
DCC to LRD transition probability	20.00%	16.00%	24.00%
HCC to LRD (sub-years) transition probability	61.00%	37.10%	66.44%
HCC to LRD (year 1) transition probability	16.20%	11.03%	23.06%
Incident MASLD risk reduction for physical activity	40%	32%	48%
Physical activity’s impact on transition rates	33%	26%	40%
Annual health care costs by disease state	See Table 1	−20%	+20%

DCC, decompensated cirrhosis; HCC, hepatocellular carcinoma; LRD, liver-related death; MASLD, metabolic dysfunction–associated steatotic liver disease.

*F0-F4 refer to fibrosis stages.

## Results

The model estimated that MASLD prevalence in Saudi Arabia would grow from 10.0 million in 2025 to 14.3 million in 2045, an increase of 42%. Over this 20-year period, an estimated 209,700 cumulative DCC cases, 28,300 cumulative HCC cases, and 159,000 liver-related deaths were projected (Table 3a). Annual health care costs were estimated at $860 million in 2025, increasing to $2.2 billion in 2045, a 160% increase. Cumulative direct health care costs were estimated at $33.2 billion during this period (Table 3b).

**Table 3:** Advanced liver disease cases and direct health care costs by scenario – Saudi Arabia, 2025-2045.

3a. Advanced Liver Disease Cases Averted by Scenario						
Scenario	Cumulative Liver Related Deaths	Cumulative Liver Related Deaths Averted	Cumulative HCC Cases	Cumulative HCC Cases Averted	Cumulative DCC Cases	Cumulative DCC Cases Averted
Status Quo	159,000	-	28,300	-	209,700	-
10% with ≥30% relative reduction in hepatic steatosis	152,200 (67,800 – 306,400)	6,800 (3,000 – 13,700)	27,000 (15,000 – 51,000)	1,400 (800 – 2,700)	199,500 (87,200 – 394,200)	10,200 (4,400 – 20,100)
20% with ≥30% relative reduction in hepatic steatosis	145,700 (64,900 – 293,400)	13,300 (5,900 – 26,800)	25,600 (14,300 – 48,600)	2,700 (1,500 – 5,200)	189,700 (82,900 – 374,900)	19,900 (8,700 – 39,500)
30% with ≥30% relative reduction in hepatic steatosis	139,500 (62,100 – 280,800)	19,500 (8,700 – 39,300)	24,300 (13,600 – 46,100)	4,000 (2,300 – 7,700)	180,400 (78,900 – 356,500)	29,300 (12,800 – 57,800)
**3b. Costs and DALYs Averted by Scenario**						
**Scenario**	**Cumulative Direct Costs (USD Millions)**	**Cumulative Costs Averted (USD Millions)**	**Cumulative DALYs Averted**			
Status Quo	$33,200	–	-			
10% with ≥30% relative reduction in hepatic steatosis	$31,900 ($16,500 - $61,300)	$1,300 ($700 - $2,500)	26,800 (13,000 – 51,500)			
20% with ≥30% relative reduction in hepatic steatosis	$30,600 ($15,900 - $58,900)	$2,600 ($1,300 - $5,000)	52,100 (25,200 – 100,000)			
30% with ≥30% relative reduction in hepatic steatosis	$29,400 ($15,200 - $56,400)	$3,800 ($1,900 - $7,300)	75,900 (36,800 – 145,500)			

DCC, decompensated cirrhosis; HCC, hepatocellular carcinoma.

DALYs, disability-adjusted life years; USD, United States dollars.

**Fig 2:**
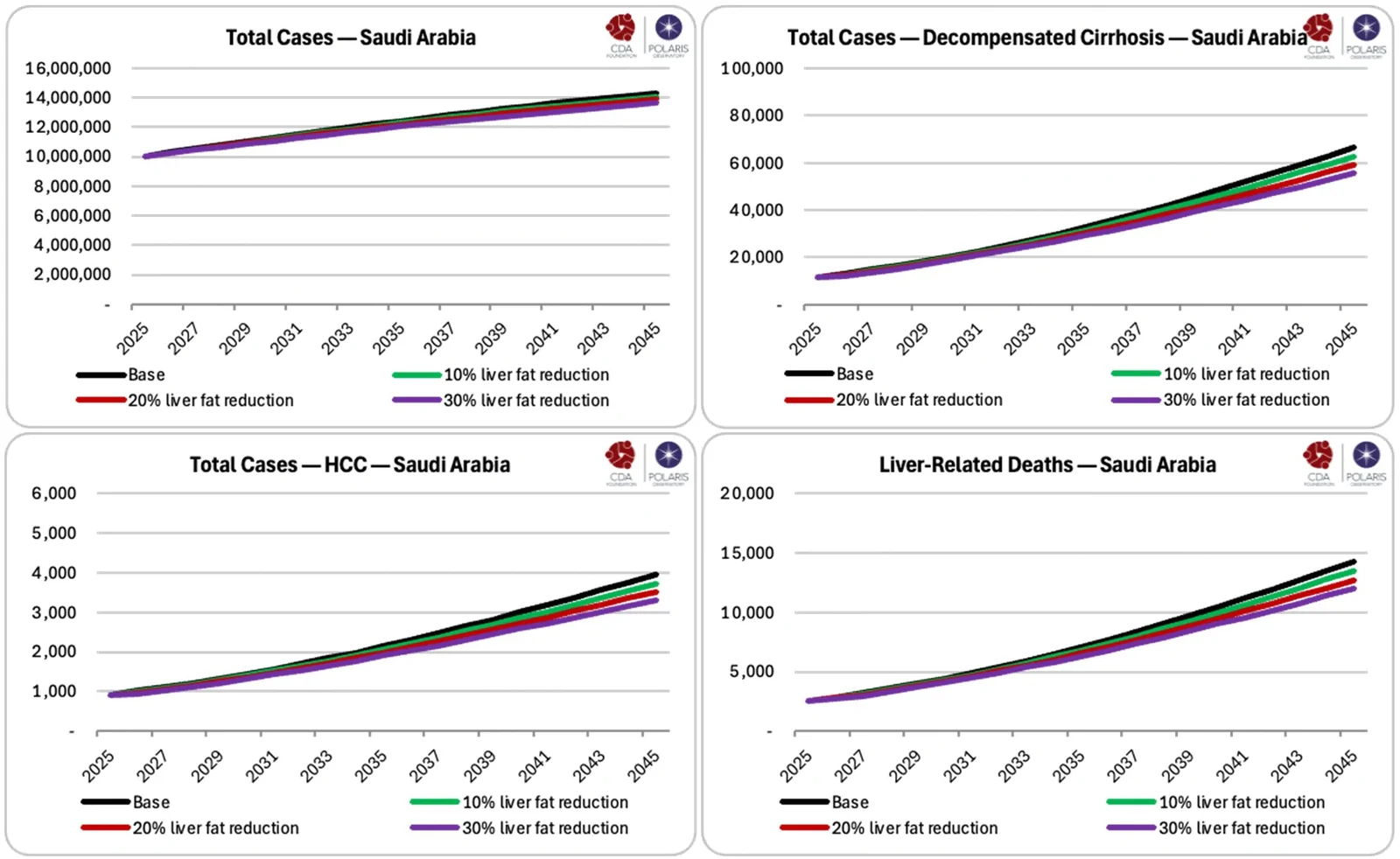
Disease burden by scenario – Saudi Arabia, 2025-2045. Scenarios depict 10%, 20%, and 30% of the MASLD population achieving the clinically significant threshold of ≥30% relative reduction in hepatic steatosis. (A) Total MASLD cases (curves represent the reduction of the number of cases under three scenarios). (B) Total decompensated cirrhosis cases (curves represent the reduction of the number of cases under three scenarios). (C) Total hepatocellular carcinoma cases (curves represent the reduction of the number of cases under three scenarios). (D) Total liver-related deaths (curves represent the reduction of the number of deaths under three scenarios).

In aspirational scenarios where 10%, 20%, and 30% of individuals with MASLD achieved ≥30% relative reduction in hepatic steatosis, total MASLD prevalence could decline by 0.5%, 1.0%, and 1.5% by 2045, a relative change of 1.5% to 4.7%. Both DCC and HCC cases could be reduced by nearly 5% to ≥14%, depending on scenario, and cumulative liver-related deaths could decline by 4.3%, 8.3%, and 12.3%, respectively (Fig 2). With 30% of individuals achieving the milestone reduction in hepatic steatosis due to increased physical activity, 19,500 liver-related deaths and 4,000 HCC cases could be prevented by 2045. Cumulative direct health care costs could be reduced by 4.0%, 7.8%, and 11.5% across the scenarios ($1.3 billion to $3.8 billion), while cumulative DALYs averted could rise from 8.6% to 24.2% across scenarios, reflecting greater health gains with increased physical activity.

**Fig 3:**
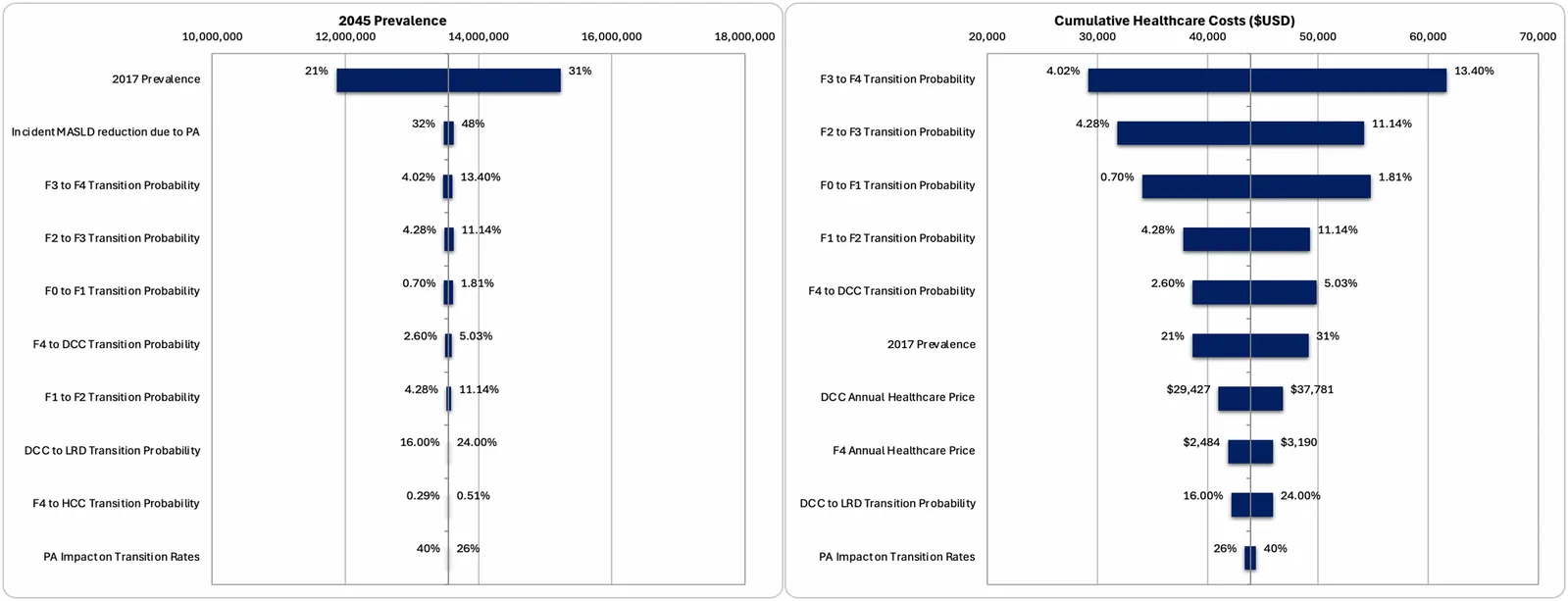
Tornado diagrams summarizing sensitivity analysis. Results from the probabilistic sensitivity analysis are summarized in the diagrams. (A) 2045 prevalence (the largest driver of uncertainty was the 2017 prevalence estimate). (B) Total health care costs, 2025-2045 (the largest drivers of uncertainty for cumulative MASLD health care costs were the transition rates between fibrosis stages).

Results from the probabilistic sensitivity analysis are reported as 95% uncertainty intervals in Table 3. Sensitivity analysis of projected 2045 prevalence showed that the largest driver of uncertainty was the prevalence estimate for 2017, accounting for 99% of the total variance. The largest drivers of uncertainty for cumulative MASLD health care costs were the transition rates between fibrosis stages, specifically F3 to F4, F2 to F3, F0 to F1, F1 to F2, and F4 to DCC, collectively accounting for 93% of total variance. Results of the sensitivity analysis are summarized as tornado diagrams in Fig 3.

## Discussion

The findings of our modeling study demonstrate that increased physical activity could substantially reduce MASLD burden in Saudi Arabia. Across model scenarios, achieving a relative reduction of ≥30% hepatic steatosis among 10% to 30% of individuals was associated with an estimated 4% to 12% reduction in direct health care costs, a 4–12% decline in liver-related deaths, and a 9–24% reduction in cumulative DALYs over the next 20 years. In addition, preventing MASLD onset through physical activity reduced the number of new cases by an estimated 254,300–762,800.

Although our analysis did not measure the specific level of physical activity, existing literature supports the plausibility of a ≥ 30% hepatic steatosis reduction threshold. For example, one study found that an increase of 2,500 steps per day resulted in a 24% lower risk of MASLD progression [33], falling within the same range as modeled MASH resolution.

The European Association for the Study of the Liver and the American Association for the Study of Liver Diseases strongly recommend physical activity in adults with MASLD [41,42]. Those who met the recommended ≥150 minutes per week were most likely to have MASH resolution, although any increase in the number of days of physical activity per week was associated with steatosis resolution [43], supporting all levels of physical activity for disease benefits. Studies have also substantiated the reaccumulation of hepatic steatosis, and hence MASLD, in those who ceased physical activity [44,45], emphasizing the importance of long-term strategies promoting physical activity. Further, increasing physical activity reduces the likelihood of developing steatosis. The protective role of physical activity is evident not only in individuals who have consistently maintained physical activity but also in those who initiate physical activity [46], again reinforcing the need to support a wide range of physical activity programs and policies to help diminish MASLD burden.

The modeling results can support Saudi Arabia’s policymakers in future evidence-driven decision making related to the implementation of Vision 2030’s Health Sector Transformation Program [12], including the preparation of program delivery plans. One objective of the program is to strengthen prevention against health threats, including noncommunicable diseases. The modeling provided additional evidence for allocating more resources to preventive interventions, targeting both high-risk populations—where physical activity supports the prevention and control of obesity—and patients with MASLD. The physical activity promotion initiatives need to be closely coordinated with the initiatives supported under the Quality of Life Program and implemented outside of the health sector [14].

This study has potential limitations. The model did not consider the effects of diet and other lifestyle modifications, nor the potential impact of glucagon-like peptide-1 receptor agonists on the incidence or prevalence of MASLD. In addition, mortality rates included in the model are based on the overall MASLD population. There is evidence that increased physical activity reduces mortality rates [47,48]. Since only a subset of the modeled MASLD population was assumed to increase physical activity levels, the overall mortality rates were not adjusted down, which may slightly overstate mortality and understate the disease and economic benefits of increased physical activity among the MASLD population. Other limitations include uncertainty in transition rates, simplified assumptions regarding behavioral uptake and sustainability of physical activity, and reliance on U.S.-based health care cost estimates (adjusted for inflation) due to the lack of Saudi Arabia–specific cost data by MASLD disease stage. Differences in health care pricing, reimbursement mechanisms, and patterns of care between the United States and Saudi Arabia may affect the accuracy of the estimated economic burden.

Nonetheless, strengths of the analysis include the use of validated progression models, Saudi-specific prevalence data, and integration of both health and economic outcomes. This analysis also captured the effect of physical activity on both MASLD incidence and progression. To our knowledge, this is the first study to quantify improvements in MASLD disease outcomes and health care costs directly associated with physical activity. While this analysis focuses on Saudi Arabia, the model framework and findings can be applicable to other countries with high MASLD prevalence and insufficient physical activity levels.

In conclusion, insufficient physical activity contributes to the rising MASLD burden in Saudi Arabia. The findings reinforce the need to include MASLD prevention in physical activity promotion campaigns and noncommunicable disease advocacy efforts. Promoting physical activity at the population level can play a significant role in reducing the health and economic burden of this rapidly growing disease.

## Supporting information

S1 File Supporting information on the modeling approach. The Supporting File 1 provides further information on modeling, including the Markov model and equations used, calculation of transition rates, and estimations of MASLD incidence and prevalence.
(DOCX)

S2 File The values used to build figures. The Supporting File 2 provides (i) a minimum dataset used for modeling and underlying the results of the study and (ii) data points used to prepare Figure 2 and (iii) data points used to prepare Figure 3.
(XLSX)

## Abbreviations

AASLD: American Association for the Study of Liver Diseases
CVD: cardiovascular disease
DCC: decompensated cirrhosis
DALYs: disability-adjusted life years
EASL: European Association for the Study of the Liver
GDP: gross domestic product
HCC: hepatocellular carcinoma
MASH: metabolic dysfunction-associated steatohepatitis
MASLD: metabolic dysfunction-associated steatotic liver disease
NAS: non-alcoholic fatty liver disease activity score
NASH: non-alcoholic steatohepatitis
RCT: randomized controlled trial
UK: United Kingdom
USD: United States dollars
