# Supplementary material for: Modelling the impact of physical activity on MASLD in Saudi Arabia
Source: PLoS One. 2026 Sep 15;21(9):e0354332. doi: 10.1371/journal.pone.0354332 (PMC13577342; doi:10.1371/journal.pone.0354332)
Supplement: Supplementary file 1 [file pone.0354332.s001.docx]

**Supporting information on the modeling approach**

# 1. Markov model

The Markov model was built using Microsoft Excel^®^ (Microsoft Corp., Redmond, WA) to track the annual population with the metabolic dysfunction–associated steatotic liver disease (MASLD) by fibrosis stage and the metabolic dysfunction-associated steatohepatitis (MASH) status (steatosis only [MASL] or MASH) from 1950-2050.

Beginning with the estimated annual new MASLD cases (defined as the onset of steatosis rather than newly diagnosed), fibrosis progression of all cases was modeled through 2050. Cases by stage of disease were calculated annually by age and gender, with one-year age cohorts through age 84 and cases aged ≥85 years tracked as a single cohort. Annually, the population in each age group (excluding the ≥85 year cohort) was advanced to the next age to simulate the impact of aging. Historical and medium-fertility projection population data were obtained from the United Nations’ population database by gender and one year age cohort [1].

Disease progression through fibrosis and advanced liver disease (decompensated cirrhosis and HCC) was estimated with adjustment for all-cause mortality (including general background, excess cardiovascular and liver-related mortality). New cases by disease stage (*New Cases* _stage x_) were calculated by multiplying progression rates and the total cases at prior stages of the disease in the previous year (*Total Cases* _stage x-1, Year Y-1_) as shown in Equation 1.

Equation 1

$${Total Cases}_{{Stage}_{x} \& {Year}_{y} \& {Age Cohort}_{z}}=\left( {Total Cases}_{{Stage}_{x} \& {Year}_{y-1} \& {Age Cohort}_{z-1}} \right)+ {New Cases}_{{Stage}_{x} \& {Year}_{y} \& {Age Cohort}_{z}}-{All Cause Mortality}_{{Stage}_{x} \& {Year}_{y} \& {Age Cohort}_{z}}-{Progressed}_{{Stage}_{x} \& {Year}_{y} \& {Age Cohort}_{z}}$$

where:

${{New Cases}_{{Stage}_{x} \& {Year}_{y} \& {Age Cohort}_{z}}= (Total Cases}_{{Stage}_{x-1} \& {Year}_{y-1} \& {Age Cohort}_{z}})({Progression Rate}_{{Stage}_{x-1}\to{Stage}_{x} \& {Age Cohort}_{z}}$)

${Background Mortality}_{{Stage}_{x} \& {Year}_{y} \& {Age Cohort}_{z}}= ({Total Cases}_{{Stage}_{x} \& {Year}_{y-1} \& {Age Cohort}_{z}})([Background {Mortality Rate][CVD Multiplier]}_{{Age Cohort}_{z}}$)

${{Progressed}_{{Stage}_{x} \& {Year}_{y} \& {Age Cohort}_{z}}= (Total Cases}_{{Stage}_{x-1} \& {Year}_{y-1} \& {Age Cohort}_{z}})({Progression Rate}_{{Stage}_{x}\to{Stage}_{x+1} \& {Age Cohort}_{z}}$)

$${Liver Related Mortality}_{\mathrm{Stage}_{x} \mathrm{Year}_{y} {Age Cohort}_{z}}=\left( {Total Cases}_{\mathrm{Stage}_{x} \mathrm{Year}_{y-1} {Age Cohort}_{z-1}}-{Adjusted Background Mortality}_{\mathrm{Stage}_{x} \mathrm{Year}_{y} {Age Cohort}_{z}}-\mathrm{Progressed}_{\mathrm{Stage}_{x} \mathrm{Year}_{y} {Age Cohort}_{z}} \right)\left( Liver Related {Mortality Rate}_{{\mathrm{Yea}r_{y-1}Age Cohort}_{z-1}} \right)$$

# 2. Transition rates

The annual transition probabilities were based on published estimates and expert consensus, as shown in Table S1. However, single annual transition rates resulted in poor validation of the models (i.e., comparison of modeled and reported hepatocellular carcinoma (HCC) cases). Thus, age- and gender-specific fibrosis progression rates were developed based on assumptions for the distribution of cases by MASH status and fibrosis stage, as described below. Fibrosis progression rates are available from studies analyzing consecutive liver biopsies, but report highly varied rates, including negative progression (e.g. regression) [2]. For the purpose of the model, progression rates were assumed to be the sum of forward progression minus the rate of regression. Where data or expert input were available for the incidence of MASLD-related HCC, decompensated cirrhosis and related mortality, progression rates were modified to align with reported data and expert consensus (Table S1). A long-term follow-up study of individuals with MASH-related cirrhosis reported that 45% experienced liver failure or decompensated cirrhosis, defined as an increase in Child-Turcotte-Pugh score by 2 points over twelve years of follow-up in patients with Child Class A Cirrhosis [3]. An annual progression rate of 3.8% decompensation among cirrhotics was calculated and applied in the model.

Fibrosis progression rates were further adjusted with overweight individuals (BMI 25 to <30kg/m^2^) having 2.35 times greater odds and obese individuals (BMI ≥30kg/m^2^) having 5.70 times greater odds of advanced fibrosis [4]. It was assumed that relative differences in the proportion of overweight and obese individuals would be reflective of the country’s MASLD population.

It was assumed that 64% of incident HCC cases would occur among cirrhotics [5]. Using U.S. data, the annual transition rate from F4 to HCC was estimated at 0.38%. The remaining 36% of incident HCC cases occurred among F0-F3 cases. The incidence rate among F3 cases was back calculated and progression decreased exponentially with each decreasing level of fibrosis from 0.03% (F3 to HCC) to 0.0004% (F0 to HCC) (Table S1). MASLD-related HCC cases may experience greater mortality as compared to HCV-related HCC; first year mortality (61%) was applied to new HCC cases, with subsequent years mortality rates based on long-term survival data [6,7]. The annual calculated transition rates are shown in Table S2. Transition rates adjusted for the effect of physical activity are shown in Table S3.

Table S1. Disease Stage Annual Transitions Rates

| Disease Stage Transition | Annual Rate | Source |
| --- | --- | --- |
| F0 to F1 | 0.3-2.2% | Back-calculated |
| F0 to HCC | 0.0004% | [5,6] |
| F1 to F2 | 2.8-13.3% | Back-calculated |
| F1 to HCC | 0.009% | [5,6] |
| F2 to F3 | 2.8-13.3% | Back-calculated |
| F2 to HCC | 0.02% | [5,6] |
| F3 to F4 (Cirrhosis) | 3.8-9.9% | Back-calculated |
| F3 to HCC | 0.03% | [5,6] |
| Cirrhosis to Decomp Cirrhosis | 3.80% | [3] |
| Cirrhosis to HCC | 0.38% | [5,6] |
| Decomp Cirrhosis to Liver Rel. Death | 20.0% | [3] |
| HCC to Liver Rel. Death (Year 1) | 61.0% | [6] |
| HCC to L.R. Death (Sub Years) | 16.2% | [7] |

Table S2. Fibrosis Transition Probabilities by Disease Stage, Sex and Age Group – Saudi Arabia

Table S3. Fibrosis Transition Probabilities by Disease Stage, Sex and Age Group – Saudi Arabia –
Adjusted for the Effect of Physical Activity

# 3. Incidence and prevalence

Recent and accurate estimates of MASLD incidence and prevalence were either unavailable, had limitations that precluded application to the general population, or were subject to varied diagnostic techniques. Therefore, annual changes in the number of new cases were back calculated using the change in obesity as a surrogate for the change in new MASLD cases. Total prevalent cases were assumed to be the sum of existing and new MASLD cases after accounting for mortality and were calibrated to the estimated prevalence of MASLD in 2015. Incidence was used to describe new MASLD cases (onset of steatosis) and not the time of first diagnosis.

The reported rates of adult obesity and diagnosed diabetes have increased over time [8,9]. Unlike obesity, Type 2 Diabetes Mellitus (T2DM) data are less readily available due to changes in awareness, screening, and diagnosis levels. Reliable estimates of true T2DM prevalence (diagnosed and undiagnosed) were unavailable until recent decades. Long-term changes in adult obesity were plotted [8,10-12]. The growth in MASLD new cases was assumed to follow the growth in obesity. Future trends in adult obesity were forecasted using best-fit sigmoidal functions.

Published data suggest that males have a higher MASLD prevalence than females and prevalence rates increase with age [13-15]. Relative incidence values describe changes in the annual number of new MASLD cases. A curve was fitted from 1950 to the estimated peak and a second curve followed the decline in relative incidence. Relative changes in the number of total MASLD cases and the distribution of MASL versus MASH within the population were imputed from data related to trends for obesity for which more robust data existed.

Annual relative incidence values were used to describe changes in the annual number of new MASLD cases over time. The Excel® Solver add-in was used to solve for the constant, which when multiplied by the annual relative incidence, resulted in the known prevalence after adjusting for mortality. This constant multiplied by the relative incidence provided the number of new MASLD cases per year. Data related to the distribution of MASL vs. MASH in these populations were used to impute the trends for these histological phenotypes [15-17].

Next, annual incident cases were distributed by age and gender to fit the adjusted MASLD prevalence. A weighting factor was applied to reported prevalence by age and gender to reach estimated MASLD prevalence in the adult age groups in 2025 (Table S4). The percentage of the incident population allocated to each age and gender cohort in years 1950-1965 was set equal to 1966 and trended linearly in five-year increments until 2011, at which point the percent of incident cases allocated to each age and gender cohort were held constant until 2050. The relative impact of incident MASLD cases occurring prior to 1950 was negligible and not included.

# Table S4. Prevalence of MASLD by Stage – Model Forecasts

| **Stage** | **2025** | **2035** | **2045** |
| --- | --- | --- | --- |
| F0 | 8,463,300 | 9,865,300 | 10,857,200 |
| F1 | 793,900 | 1,068,100 | 1,259,000 |
| F2 | 424,900 | 684,600 | 892,200 |
| F3 | 237,300 | 472,200 | 714,100 |
| F4 | 114,400 | 282,200 | 516,800 |
| Advanced Liver Disease | 13,800 | 36,900 | 72,600 |
| Total | 10,047,500 | 12,409,300 | 14,311,900 |
| Prevalence % | 29.1% | 31.0% | 31.7% |

Advanced Liver Disease includes DCC, HCC, and Liver Transplantations.

# References

1.	United Nations, Department of Economic and Social Affairs, Population Division. World Population Prospects 2024. United Nations; 2024. Database [Internet]. Available from: https://population.un.org/wpp/.

2.	Singh S, Allen AM, Wang Z, Prokop LJ, Murad MH, Loomba R. Fibrosis progression in nonalcoholic fatty liver vs nonalcoholic steatohepatitis: a systematic review and meta-analysis of paired-biopsy studies. Clin Gastroenterol Hepatol. 2015; 13(4): 643-54.e1-9; quiz e39-40.

3.	Sanyal AJ, Banas C, Sargeant C, Luketic VA, Sterling RK, Stravitz RT, et al. Similarities and differences in outcomes of cirrhosis due to nonalcoholic steatohepatitis and hepatitis C. Hepatology. 2006; 43(4): 682-9.

4.	Angulo P, Hui JM, Marchesini G, Bugianesi E, George J, Farrel GC, et al. The NAFLD fibrosis score: a noninvasive system that identifies liver fibrosis in patients with NAFLD. Hepatology. 2007; 45(4): 846-54.

5.	Rahman RN, Ibdah JA. Nonalcoholic fatty liver disease without cirrhosis is an emergent and independent risk factor of hepatocellular carcinoma: A population based study. Hepatology. 2012; 56: 241A.

6.	Younossi ZM, Otgonsuren M, Henry L, Venkatesan C, Mishra A, Erario M, et al. Association of nonalcoholic fatty liver disease (NAFLD) with hepatocellular carcinoma (HCC) in the United States from 2004 to 2009. Hepatology. 2015; 62(6): 1723-30.

7.	Ries L, Young G, Keel G, et al. *SEER survival monograph: Cancer survival among adults: U.S. SEER program, 1988-2001, patient and tumor characteristics*. National Cancer Institute, SEER Program; 2007. <http://www.seer.cancer.gov>

8.	NCR Risk Factor Collaboration (NCD-RisC). Worldwide trends in underweight and obesity from 1990 to 2022: a pooled analysis of 3663 population-representative studies with 222 million children, adolescents, and adults. Lancet. 2024; 403(10431): 1027-50.

9.	NCD Risk Factor Collaboration (NCD-RisC). Worldwide trends in diabetes since 1980: a pooled analysis of 751 population-based studies with 4.4 million participants. Lancet. 2016; 387(10027): 1513-30.

10.	Hales CM, Carroll MD, Fryar CD, Ogden CL. Prevalence of obesity and severe obesity among adults: United States, 2017-2018. NCHS Data Brief. 2020; (360):1-8.

11.	NCD Risk Factor Collaboration (NCD-RisC). Trends in adult body-mass index in 200 countries from 1975 to 2014: a pooled analysis of 1698 population-based measurement studies with 19.2 million participants. Lancet. 2016; 387(10026): 1377-96.

12.	NCD Risk Factor Collaboration (NCD-RisC). Worldwide trends in body-mass index, underweight, overweight, and obesity from 1975 to 2016: a pooled analysis of 2416 population-based measurement studies in 128.9 million children, adolescents, and adults. Lancet. 2017; 390(10113): 2627-42.

13.	Fan JG, Farrell GC. Epidemiology of non-alcoholic fatty liver disease in China. J Hepatol. 2009; 50(1): 204-10.

14.	Caballeria L, Pera G, Auladell MA, Toran P, Munoz L, Miranda D, et al. Prevalence and factors associated with the presence of nonalcoholic fatty liver disease in an adult population in Spain. Eur J Gastroenterol Hepatol. 2010; 22(1): 24-32.

15.	Lazo M, Hernaez R, Eberhardt MS, Bonekamp S, Kamle I, Guallar E, et al. Prevalence of nonalcoholic fatty liver disease in the United States: the Third National Health and Nutrition Examination Survey, 1988-1994. Am J Epidemiol. 2013; 178(1): 38-45.

16.	Younossi ZM, Koenig AB, Abdelatif D, Fazel Y, Henry L, Wymer M. Global epidemiology of nonalcoholic fatty liver disease-Meta-analytic assessment of prevalence, incidence, and outcomes. Hepatology. 2016; 64(1): 73-84.

17.	Portillo-Sanchez P, Bril F, Maximos M, Lomonaco R, Biernacki D, Orsak B, et al. High Prevalence of Nonalcoholic Fatty Liver Disease in Patients With Type 2 Diabetes Mellitus and Normal Plasma Aminotransferase Levels. *J Clin Endocrinol Metab.* 2015; 100(6): 2231-8.
